# 1,25-Dihydroxyvitamin D3 Promotes a Sustained LPS-Induced NF-κB-Dependent Expression of CD55 in Human Monocytic THP-1 Cells

**DOI:** 10.1371/journal.pone.0049318

**Published:** 2012-11-12

**Authors:** Michael G. Izban, Bogdan J. Nowicki, Stella Nowicki

**Affiliations:** 1 Department of Microbiology and Immunology, Meharry Medical College, Nashville, Tennessee, United States of America; 2 Department of Obstetrics and Gynecology, Meharry Medical College, Nashville, Tennessee, United States of America; University of Tennessee, United States of America

## Abstract

The vitamin D3 system imposes immunosuppressive effects on monocytic cells, in part, by inhibiting NF-κB-dependent expression of proinflammatory mediators. CD55, a cell surface complement regulatory protein that promotes protective and anti-inflammatory properties, is reportedly an NF-κB target gene transiently induced in monocytic cells by the bacterial endotoxin LPS. CD55 is elevated on white cells in women experiencing preterm labor (a pathophysiology commonly associated with bacterial infection) and failure to maintain CD55 was associated with subsequent preterm delivery. We examined the influence of vitamin D3 signaling on LPS-induced expression of CD55 in human monocytic THP-1 cells using quantitative PCR, immunoblot, immunohistochemistry, and NF-κB activation pathway inhibitors. Non-NF-κB targets CD14 and CD11b, which modulate bacterial surveillance and eradication, respectively, were also examined. LPS produced a rapid transient 1.6-fold increase in CD55 mRNA. 1,25-D3 alone did not affect CD55 mRNA expression within the first 48 h. However, in 1,25-D3 pretreated cells, LPS produced a >4-fold immediate and sustained increase in CD55 mRNA and protein expression, which was blocked by NF-κB inhibitors. Our results unexpectedly suggest that vitamin D3 signaling may promote an anti-inflammatory response through an NF-κB-dependent increase in CD55 expression. As expected, LPS or 1,25-D3 alone led to sustained increases in CD14 and CD11b expression. In 1,25-D3 pretreated cells, LPS differentially regulated protein expression - CD14 (21-fold increase) and CD11b (a transient 2-fold decrease) - principally at the posttranscriptional level. The coordinated temporal expression of CD55, CD14 and CD11b would contribute to an anti-inflammatory response by providing protection against complement-mediated cell lysis during pathogen recognition and eradication. Overall, the vitamin D3 system may play a role coordinating an anti-inflammatory response pattern of the host complement immune system. This may be particularly important when considering the high rates of preterm births in blacks, a population that exhibits reduced circulating vitamin D3 levels.

## Introduction

Inappropriate or excessive activation of the complement system contributes to the pathophysiology of many human inflammatory and autoimmune diseases, such as rheumatoid arthritis, cardiovascular disease [1] and the pathophysiology of allograft rejection [2] and pregnancy [3]. All serum exposed cells express cell surface complement regulatory proteins such as CD55 [4]. However, the molecular mechanism(s) by which CD55 expression is regulated during the inflammatory response remain largely unexplored. CD55 is a key regulator affecting all three complement activation pathways and can display a net anti-inflammatory role working via several different mechanisms. It intrinsically dissociates (or prevents) the association of C3/C5 convertases that assemble on the cell surface thereby blocking cell surface complement activation and subsequent formation of lytic membrane attack complexes [5]. CD55-mediated inhibition of complement activation downstream of the C3 component allows it to act as an anti-inflammatory mediator by preventing the production of soluble C3a and C5a, thereby regulating the induction of local and systemic inflammatory responses [2], [6], [7]. CD55 is also an immunological anti-adhesive molecule implicated in the resolution of ongoing inflammation of mucosal epithelia through clearance of transmigrating neutrophils [8].

Depending on the temporally regulated balance of local pro- and anti-inflammatory mediators, monocytic cell lineages participate in a variety of seemingly disparate physiological processes including innate and adaptive immuno-surveillance and tissue repair and remodeling [9]. Both their dynamic temporal responsiveness to pathogens and locally produced mediators, and their ability to either exacerbate or attenuate diseases, make monocytes attractive therapeutic targets [10]. Several receptors, such as the β_2_-integrin complement receptor (CR) 3 (α_M_β_2_, CD11b/CD18) and CD14, whose expression is elevated on mature monocytes and macrophages, play key roles in acute inflammatory signaling, the innate eradication of infection, and clearance of cellular debris. As such, they lead toward the resolution of inflammation [11]–[13]. CR3 influences cellular migration and mediates internalization of iC3b and non-opsonized particles [14], [15]. The LPS binding protein CD14 is a co-regulator for innate immune pathogen-associated molecular pattern recognition signal transduction receptors and functions as a sensitivity rheostat in pathogen surveillance [16]. CD14 also plays a role in CR3-dependent phagocytosis [11], [13]. While the early stages of pathogen eradication and/or clearance of damaged human cells are essential components in the resolution of inflammation, inappropriate and excessive activation of the complement system may lead to injury of bystander intact tissue and a chronic pro-inflammatory response. Therefore, sufficient expression of anti-inflammatory CD55 is necessary to balance complement-mediated pro-inflammatory responses.

Our previous studies demonstrated that CD55 expression is elevated in peripheral leukocytes of women with preterm labor (PTL); further all PTL subjects diagnosed with urogenital infections exhibited elevated levels of CD55 [17], [18]. We speculated that the upregulation of CD55 may occur in response to the bacterial lipopolysaccharide (LPS) endotoxin. LPS principally leads to a transient activation of NF-κB [19], a major signal transduction molecule utilized in the regulation of proinflammatory immune responses [20]. However, LPS signaling directly and indirectly orchestrates a complex and time dependent gene expression program in monocytic cell lineages [21]. For example, while neither CD14 nor CD11b are NF-κB target genes [22], LPS induces their expression in monocytic cell types. Upregulation of CD55 expression has been demonstrated using primary cultures of heterogeneous populations of human peripheral leukocytes [23] or monocyte-derived dendritic cells [24] after 24 or 48 hours of LPS exposure, respectively. A genome-scale promoter survey identified CD55 as a potential NF-κB target gene and demonstrated, using human primary macrophage and microchip analysis, that CD55 mRNA pools exhibited an early 2 h transient increase in size, followed by a later increase at 24 h [22]. Whether NF-κB activation is sufficient to promote enhanced sustained CD55 expression in monocytic cell lineages is unknown.

Biologically active 1,25-dihydroxyvitamin D3 (1,25-D3) regulates not only calcium homeostasis, but also the immune functions of defense and self-tolerance [25]. In response to LPS, monocytic cells likely contribute to the local production of 1,25-D3 at sites of inflammation and 1,25-D3 activates an antimicrobial pathway in human macrophages [26]. Moreover, direct and indirect effects of local 1,25-D3 production at the site of inflammation is implicated in the promotion of anti-proliferative and anti-inflammatory processes necessary for resolution of inflammation [25]. The anti-inflammatory properties of 1,25-D3 in monocytic lineages are due, in part, to an 1,25-D3-dependent modulation of NF-κB signaling components that reduce the level of NF-κB activation [27], [28]. The immunomodulatory effects of 1,25-D3 on CD55 expression in monocytic cells is unknown. The vitamin D3 system is also augmented throughout pregnancy as uterine decidua and placental tissues convert circulating 25-D3 into 1,25-D3 [29], [30]. Recent studies suggest that vitamin D deficiency is common during pregnancy, especially in dark skinned women, and that alterations in vitamin D3 metabolism and signaling may be independent risk factors for preeclampsia [31], fetal growth restriction [32] and preterm delivery [33].

In this study we systematically examine the effects of 1,25-D3, LPS, and LPS following 1,25-D3 pretreatment on the temporal expression of CD55, CD14 and CD11b in human-derived THP-1 monocytes. We show that 1,25-D3 pre-treatment differentially regulated the LPS-induced temporal expression patterns of all three molecules. 1,25-D3 pretreatment altered the LPS-induced expression of CD55 from a transient to a sustained increase and we provide compelling evidence that LPS-induced expression of CD55 is functionally dependent on NF-κB activation. Thus, 1,25-D3 influences monocytic response to pro-inflammatory mediators, such as LPS, by an NF-κB-dependent activation of the complement regulatory protein CD55. These findings strongly support the notion that the NF-κB signaling pathway directly promotes an anti-inflammatory response by limiting complement activation. Our systematic studies also demonstrate that 1,25-D3 influences the LPS-induced expression of CD14 and CD11b and that post-transcriptional mechanisms are likely to play an important regulatory role in modulating their steady state levels.

These findings have special significance because they suggest a direct molecular integration of the complement and vitamin D3 hormonal systems, both of which are linked to chronic inflammatory and infectious diseases such as arthritis, cardiovascular disease, diabetes, gestational infection and pregnancy outcomes. This may be particularly important when considering health disparities and the high rates of preterm births in blacks, a population that exhibits reduced circulating vitamin D levels.

## Materials and Methods

### Cell Culture

THP-1 cells were purchased from the ATCC (Manassas, VA). They were cultured in RPMI-1640 media supplemented with 10% FBS, 2 mM glutamine, 1 mM sodium pyruvate, 4.5 g/l glucose, 10 U/ml penicillin/10 µg/ml streptomycin and 0.5 µg/ml Plasmocin (InvivoGen, San Diego CA) in an atmosphere of 5% CO_2_ and 95% humidity. All cells were mycoplasma free and the culture media tested negative for endotoxins using the Pyrotell Limulus Amebocyte lysate test (Associates of Cape Cod Inc., East Falmouth, MA). For time course studies, a single pool of cells was resuspended in fresh media containing vehicle (0.01% ethanol) or 100 nM 1,25-dihydroxyvitamin D3 (Calbiochem). To minimize media exhaustion, 0.5 million cells were seeded into 10 cm dishes in a total volume of 8 ml. After 72 h, 100 µl of fresh media containing vehicle (0.1% PBS) or Escherichia coli-derived LPS (0111.B4, Sigma) was added to the cells to a final LPS concentration of 1 µg/ml. Following incubation, adherent and nonadherent cells were harvested after mechanically dislodging any that had attached to the culture dish. Cells were divided in half and concentrated by centrifugation. For mRNA studies, one of the cell pellets was immediately solubilized in RNA lysis solution (RNA easy Plus, Qiagen, Valencia, CA) and RNA was purified following the manufacturer’s protocol. The matching pellet was washed once in PBS containing a Complete Protease Inhibitor Cocktail (Roche) as recommended by the manufacturer, and reconcentrated. The cell pellet was then either snap frozen in liquid nitrogen and stored at −80°C, or immediately solubilized in SDS-PAGE loading buffer. When the cumulative culture times were 96 h or less, 4.0 million cells were seeded into 10 cm dishes in a total volume of 8 ml. For the NF-κB inhibition studies 1,25-D3-primed cells were treated with either 50 µM MG132 (Sigma-Aldrich) or vehicle (0.1% DMSO) or 30 µM parthenolide (Sigma-Aldrich) or vehicle (0.1% ethanol) for 1 h prior to LPS challenge and harvested as described above after an additional 2 h or 24 h incubation.

### mRNA Pool Size Quantification

For mRNA pool size determinations, total RNA (600 ng in 25 µl) was reverse transcribed using a mixture of random and oligo(dT) primers (qScript, Quanta Biosciences, Gaithersbury MD) and following the manufacturer’s protocol. mRNA pool sizes were determined by quantitative real time PCR (qPCR) using the PerfectaCTa SYBR Green Fastmix system (Quanta Biosciences). Forty (or 45, when determining CD14 levels) amplification cycles (15 s at 95°C and 1 min at 60°C) were used. The reaction mixture contained 1.5 µl (or 1.5 µl of a 1∶500 dilution for 18S rRNA determination) of reverse transcribed RNA in a 20 µl reaction volume. PCR efficiencies and relative copy numbers were determined using the absolute standard curve method. All qPCR mRNA primers were empirically designed using VectorNTI v5.0.3 software (InforMax, Inc., Grand Island, NY) and were mRNA specific. No detectable products were generated after a 40 cycles PCR reaction containing 150,000 copies of genomic DNA. The primer sets were: CD55, f5′-ACCAAATGCTCAAGCAACACGGAG and r5′-TACTACGCTCCCAAGCAAACCTGT; CD14, f5′′- GGGACTTGGATTTGGTGGCA and r5′-CTTCGGCTGCCTCTTATATCCC; CD11b f5′- GTGAAGCCAATAACGCAGC and r5′-CTCCCATCCGTGATGACAAC; and 18s rRNA, f5′-CGGACAGGATTGACAGATTGATAGC and r5′-TGCCAGAGTCTCGTTCGTTATCG. All primers sets were visually tested for specificity and their products cloned and sequenced. All PCR efficiencies were >93%. Quantification of qPCR was performed using the BioRad icycler software. To approximate relative expression levels of CD14, CD55 and CD11b mRNA pools, a reagent-matched single run qPCR was performed.

### Confocal Microscopy

For immunofluorescence analysis, cells were seeded on glass cover slips in 24-well tissue culture plates and incubated for 72 h with 1,25-D3 and challenged with LPS for 24 h. Following removal of media, cells were fixed with freshly prepared 4% paraformaldehyde/PBS (Electron Microscopy Sciences, Hatfield, PA) for 5 min at room temperature and then stored at 4°C for 5 h. After washing with PBS, cells were blocked with blocking buffer (LI-COR). Primary antibodies (1∶250 in blocking buffer with 0.1% Tween 20) were incubated with cells overnight at 4°C. Cells were counter stained with Alexa Fluor 488 dye-conjugated secondary antibodies (Invitrogen) at room temperature (1∶500 dilution in blocking buffer with 0.1% Tween-20) for 1 h. After washing with TBS, the cells’ nucleic acids were stained using 0.5 ml of a 1 µM solution of the cell-permeant Syto 61 dye (Invitrogen), washed and the cover slips were slide mounted in Slowfade Gold antifade reagent (Invitrogen). Images were captured using a Nikon TE1000-U C1 confocal laser scanning microscope at identical settings using a 60×1.4 NA lens. Fluorescence was quantified using Nikon NIS-Elements v3.0 software.

### Immunoblotting

Immunoblot detection used the Odyssey Infrared Imaging System (LI-COR Biosciences). Cells pellets were resuspended in Laemmli buffer (25 µl/10^6^ cells) and maintained at 85°C while vortexing with an orbital mixing frequency of 1400 rpm for 15 min. The cooled mixtures were sonicated and insoluble material was pelleted. Protein concentration was determined using the Bradford method (Bio-Rad). The amount of whole cell extracted protein resolved by 8% SDS-PAGE was typically 50 or 100 µg. Prior to transfer onto an Immobilon-FL PVDF membrane (Millipore), the gel was equilibrated with two changes of transfer buffer (48 mM Tris, pH 9.2, 39 mM glycine, 0.05% SDS). Transfer was performed in the cold overnight at 25 mV. The membrane was rinsed twice with PBS prior to blocking using LiCor blocking buffer. All primary antibodies were from Santa Cruz Biotechnologies, Inc (Santa Cruz, CA) or R&D Systems in Minneapolis, MN (antibodies designated MAB). Incubations were performed overnight at 4°C with the inclusion of 0.1% Tween 20. Each antibody was tested individually and non-specific binding was minimal at the following dilutions: rabbit polyclonal against CD55 (SC-9156, 1∶400 dilution); goat anti-actin (SC-1615, 1∶10,000 dilution); and mouse monoclonal antibodies against CD11b (MAB16992, 1∶250 dilution) and CD14 (MAB3831, 1∶100 dilution). Secondary IRDye labeled antibodies raised in donkey (LI-COR Biosciences) were diluted in 1∶1 LiCor blocking/PBS containing 0.1% Tween 20 and staining was performed for 1 h. The IRDye 680 Ab (1∶10,000 dilution) was used to detect β-actin, whereas CD55, CD14 and CD11b were detected with IRDye 800CW (1∶5,000 dilution). The scan setting was at a 186 µm resolution with the intensity for the 700 channel set to 4.0 and the 800 channel at 6.5. Band integrated intensities were determined from the unaltered color image, using the lane function of the Odyssey software ver. 3.0.16 and normalized to β-actin. For immunoblots multiplexed for CD55, CD11b, CD14 and β-actin, reiterative probing was performed. Following the acquisition of CD14 and β-actin data, the membrane was briefly incubated with blocking buffer prior to incubation with anti-CD55 and anti-CD11b antibodies together and then further processed in the dark. The β-actin and CD14 signal was identical before and after detection of CD55 and CD11b.

### Statistical Analysis

Means±S.D. were calculated from at least three independent experiments. Statistical significance of differences was calculated using a two-tailed Student’s t test and GraphPad Prism ver. 4 (Graphpad software).

## Results

### 1,25-Dihydroxyvitamin D3 Induces a Delayed Weak Expression of CD55 and a Rapid Robust Expression of CD14 and CD11b mRNA in THP-1 Cells

The immunomodulatory effects of 1,25-D3 on the innate immune response of monocytic cells occur via multiple interconnected steps that are temporally regulated [34]. In the initial response phase, targeted gene activation by recruitment of the ligated vitamin D receptor and non-genomic 1,25-D3-induced signaling pathways (e.g., phospatidylinositol 3-kinase (PI3K) with subsequent activation of endogenous transcription factors such as CREB) modulate the transcriptional properties of early phase genes [35], [36]. Both pathways influence the expression of CD14 [37], [38]. Sustained expression of CD14 and induction of CD11b require in part, the induced expression of second stage regulators such as the transcriptional activator myeloid zinc-finger 1 (MZF-1) protein [39]. Studies using epithelial cell types showed that expression of CD55 can be induced by CREB [40] and while the CD55 promoter (−4000 to +400) lacked vitamin D3 response elements, our in silico analysis identified three potential MFZ-1 recognition sites within 300 base pairs of the transcriptional start site (data not shown). Therefore, it was reasonable to suppose that CD55 expression may increase during the early phase following 1,25-D3 stimulation.

**Figure 1:**
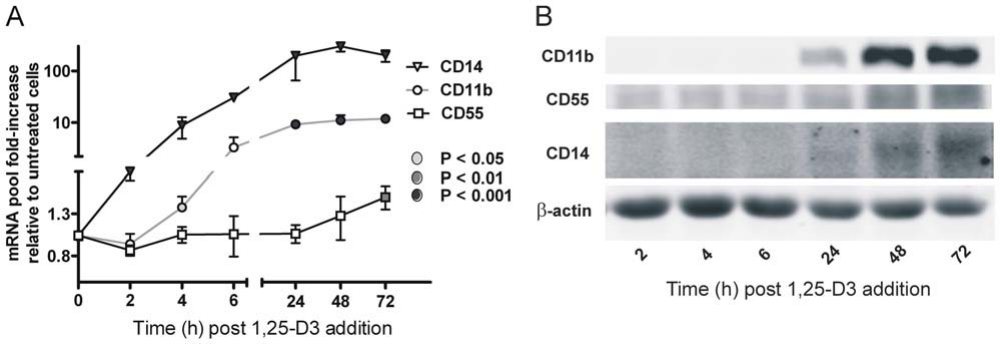
CD55 mRNA and protein expression was not altered during the early response phase (0–24 h) following a 1,25-D3 preincubation period of 72 h with human THP-1 monocytes. THP-1 cells were cultured in complete media in the absence or presence of 100 nM 1,25-D3 (added at time = 0) and harvested at the indicated times. A, CD55, CD14 and CD11b mRNA pool sizes were determined by quantitative PCR analysis using 18S rRNA as a reference. Fold-induction is expressed relative to vehicle treated controls. Values shown are the mean +/− SD of 3 independent experiments. In some instances, the SD bars are covered by the data point symbol. Shaded data points indicate statistical differences from control values. P<0.05 was considered significant. B, protein expression was analyzed in cells at various times following addition of 1,25-D3, using a fluorescence-based immunoblot approach. Corresponding controls showed no difference over the same time period.

To determine if 1,25-D3 influenced CD55 expression, THP-1 cells were grown for 72 h in culture growth media +/−100 nM 1,25-D3. This commonly used concentration of 1,25-D3 was chosen because dose-response studies report that it promotes optimal 1,25-D3-dependent effects in many cell types [41], including the monocytic THP-1 cell line and cultured primary monocytic lineages [42]–[44]. Cells were harvested at the indicated times, divided in half and processed for either quantification of CD55 mRNA (Fig. 1A) or protein expression (Fig. 1B). Effects on CD14 and CD11b were also examined. Quantitative real time PCR (Fig. 1A) demonstrated a rapid and robust increase in CD14 and CD11b mRNA pool sizes. The temporal changes in CD14 and CD11b were consistent with the proposed direct and second tier regulatory cascades, respectively, and the induced mRNA pool size was consistent with previously published studies [42], [45]. In contrast, CD55 mRNA pools were unchanged during the first 24 h of 1,25-D3 stimulation, but eventually increased 1.6-fold after 72 h. Immunoblot analysis (Fig. 1B) demonstrated a delayed increase in CD55 protein expression. CD55 protein increased <2-fold after the 24 h time point. It is unlikely that the trace pM amount of 1,25-D3 presumed present in the culture growth media [44] influences the constitutive expression of CD55 in THP-1 cells as the level of CD55 mRNA was similar when THP-1 cells were grown in culture media supplemented with charcoal-stripped fetal bovine serum (data not shown). Limitations in sensitivity precluded quantification of early changes in CD14 and CD11b protein levels, even when immunoblots contained 75 µg of cell protein. Previous reports using FACS analysis to quantify 1,25-D3-induced expression of CD14 and CD11b in THP-1 cells have shown that CD14 protein levels markedly increased within 6 h of 1,25-D3 treatment [45] and after 72 h the reported overall increase in protein expression of CD14 and CD11b (>60- and 10-fold, respectively, [39]) are consistent with the increased mRNA pool sizes seen here. These data suggest that 1,25-D3-induced early phase responses do not affect the constitutive transcriptional activity of the CD55 promoter. The modest increase in CD55 expression at 48 and 72 h following addition of 1,25-D3 is likely due to 1,25-D3-mediated changes in specific intracellular and/or autocrine signaling pathways or alterations in the nucleoprotein transcriptional machinery within the regulatory regions of the CD55 gene or both.

Finally, the CD55, CD11b and CD14 oligo sets used to measure mRNA pools have similar PCR efficiencies and our quantitative PCR analysis indicated that CD14, CD55 and CD11b had similar mRNA pool sizes (Ct values 24.8, 25.5 and 22.0, respectively) following 1,25-D3 pretreatment. Thus, the 100-fold (CD14) and 11-fold (CD11b) increase in expression elevated their mRNA pools to levels similar to that of the constitutively expressed CD55 mRNA pool. The qualitative assessment of protein expression in Fig. 1B is therefore consistent with the notion that the CD14, CD11b and CD55 protein expression levels are similar after 1,25-D3 treatment under our experimental conditions.

### Kinetics of LPS-induced Signaling and CD55, CD14 and CD11b mRNA Pool Sizes in THP-1 Cells; Differential Regulation of CD55, CD14 and CD11b

**Figure 2:**
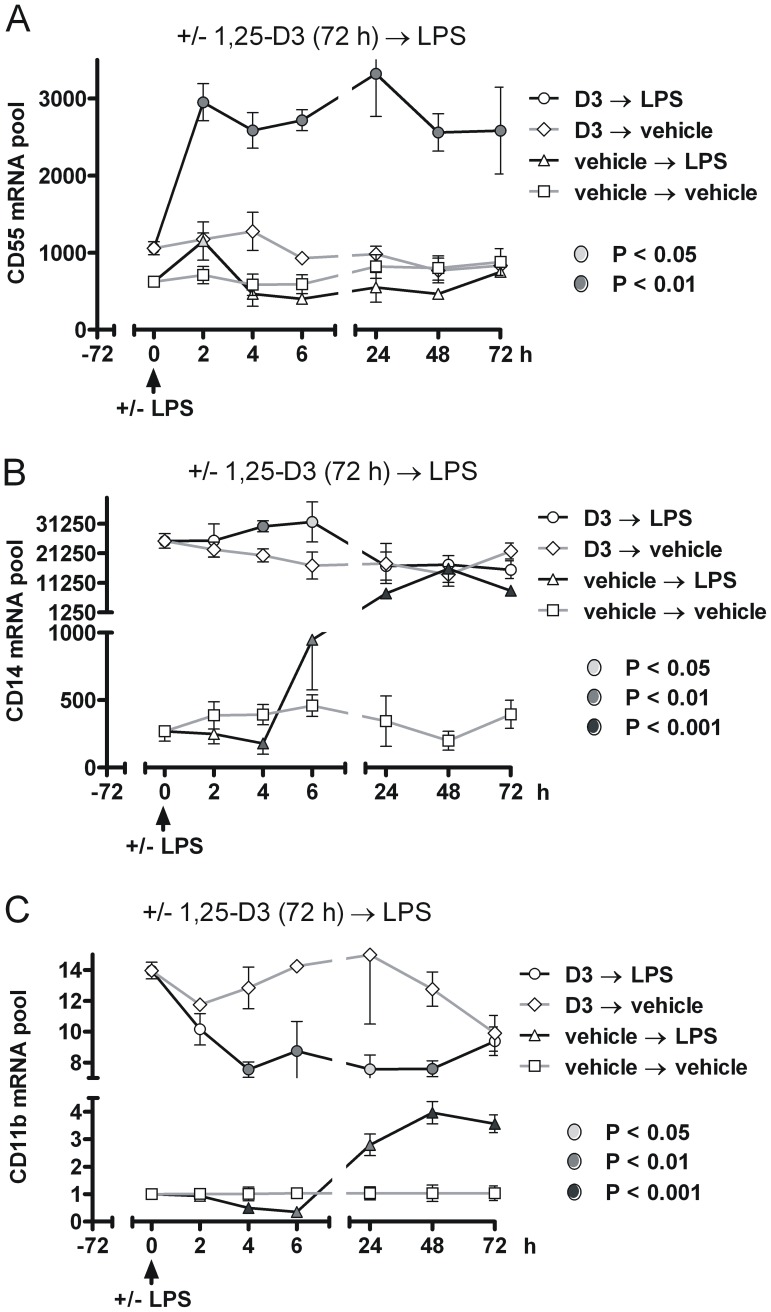
Pretreatment with 1,25-D3 for 72 h sustained the subsequent LPS-induced expression of CD55 mRNA in THP-1 cells, compared to cells pretreated with vehicle for 72 h. THP-1 cells were first preincubated in complete culture media with vehicle or with 100 nM 1,25-D3. Seventy-two h later (t = 0 on A–C), either vehicle or 1 µg/ml LPS was added and the incubation continued for an additional 72 h. Cells were harvested at the indicated times and quantitative PCR (n = 3) was used to determine CD55 (A), CD14 (B) and CD11b (C) mRNA pool sizes using the absolute copy number method. Mean levels are expressed in arbitrary units normalized to 18S rRNA (± SD). Differences in mRNA pool size were determined using a two tailed student’s t test. Shaded data points indicate statistical differences from corresponding controls.

**Figure 3:**
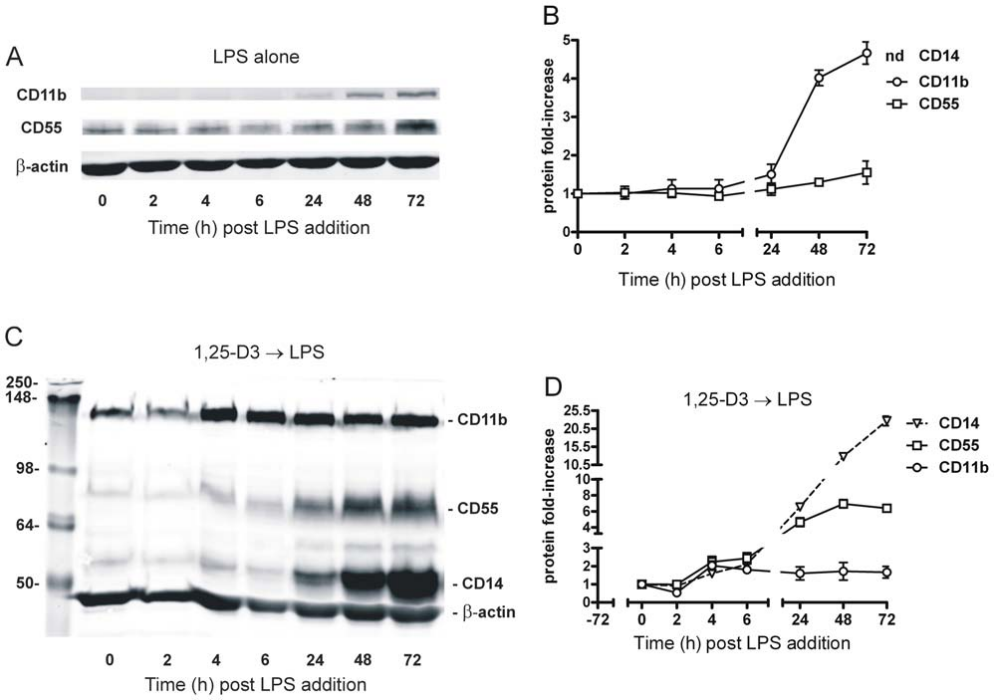
Protein expression profiles for CD55, CD14, and CD11b following addition of LPS (1 µg/ml) to THP-1 cells pretreated with or without 100 nM 1,25-D3 (72 h). A multiplexed fluorescence-based immunoblot approach was used to determine corresponding protein expression profiles from cells whose mRNA profiles are shown in Fig. 2. A, the temporal protein induction pattern of CD55, CD11b and β-actin following a LPS-challenge (without pretreatment). CD14 levels are not shown because the apparent 50-fold induction remained below the threshold of detection (nd). B, the relative band intensities for CD55 and CD11b, normalized to β-actin, are graphically presented as the average fold-change (relative to time zero) of three independent experiments (± SD). In some instances the SD are covered by the data point symbol. C and D, the multiplex immunoblot of LPS-induced protein expression in cells pretreated for 72 h with 1,25-D3 is shown in panel C; the fold induction is graphically represented in panel D as the average fold-change (relative to time zero) of three independent experiments (± SD). In some instances the SD are covered by the data point symbol. The apparent 100-fold induction of CD14 following 1,25-D3 pretreatment but before LPS (time = 0) was within the immunoblot detection limits.

We next determined the effects of LPS-induced proinflammatory signaling on CD55, CD14 and CD11b expression before and after pretreatment with 100 nM 1,25-D3 or vehicle for 72 h prior to the addition of 1 µg/ml LPS and an additional 72 h incubation period. Cells were harvested at the indicated times, divided in half and processed for either quantification of mRNA pools (Fig. 2) or protein expression (Fig. 3).

Using microchip analysis, LPS has been reported to induce transient changes in CD55 expression in human primary monocytes [22]. In THP-1 cells, quantitative PCR demonstrated a similar LPS-induced transient CD55 expression pattern which was elevated 1.6-fold following a 2 h incubation (Fig. 2A, P<0.05). Our temporal studies showed that the mRNA pools for CD14 (Fig. 2B) and CD11b (Fig. 2C) exhibited a small, transient but significant decrease at 4 h followed by a sustained increase in mRNA expression reaching 50- and 3-fold after 48 h following a LPS challenge, respectively. Immunoblot analysis for CD11b expression was consistent with the observed changes in mRNA pool sizes (Figs. 3A and B). The presumed transient increase in CD55 protein expression was undetectable and CD14 expression was below the immunoblot detection limit. Regardless, our data support the notion that LPS induced a sustained increase in CD14 and CD11b expression, albeit 2-fold (CD14) and more than 3-fold (CD11b) less than that observed following 1,25-D3 treatment.

### The LPS-induced Expression Pattern of CD55, CD11b and CD14 was Markedly Different in 1,25-D3 Pretreated Cells

As indicated above, cells grown in the 72 h preincubation period with 1,25-D3 exhibited a 1.6-fold (P = 0.008), 100-fold (P<0.001) and 13-fold (P<0.001) increase in CD55, CD14 and CD11b mRNA pools, respectively (Fig. 2A–C). However, within 2 h of an LPS challenge, the CD55 mRNA pool increased more than 3-fold (P = 0.002) and was maintained at this elevated level for the duration of the 72 h experiment. LPS also affected differentially the mRNA pool size of CD14 (Fig. 2B) and CD11b (Fig. 2C). By 6 h, the CD14 mRNA pool size had increased by 1.6-fold (P = 0.035) and returned to control levels within 24 h of LPS exposure. In contrast, CD11b mRNA pools unexpectedly decreased by a factor of 2 within 4 h of LPS addition (P = 0.002) and was maintained at this low level for 48 h.

These data demonstrate that the expression profiles of key cell surface receptor molecules involved in the innate immune surveillance (CD14) and complement (CD55 and CD11b) systems showed dramatic differences in their temporal expression patterns in response to LPS in 1,25-D3 pretreated THP-1 cells.

### CD55, but not CD14 and CD11b Protein Expression in THP-1 Cells Paralleled the Observed Changes in mRNA Pool Size in 1,25-D3 Pretreated Cells

**Figure 4:**
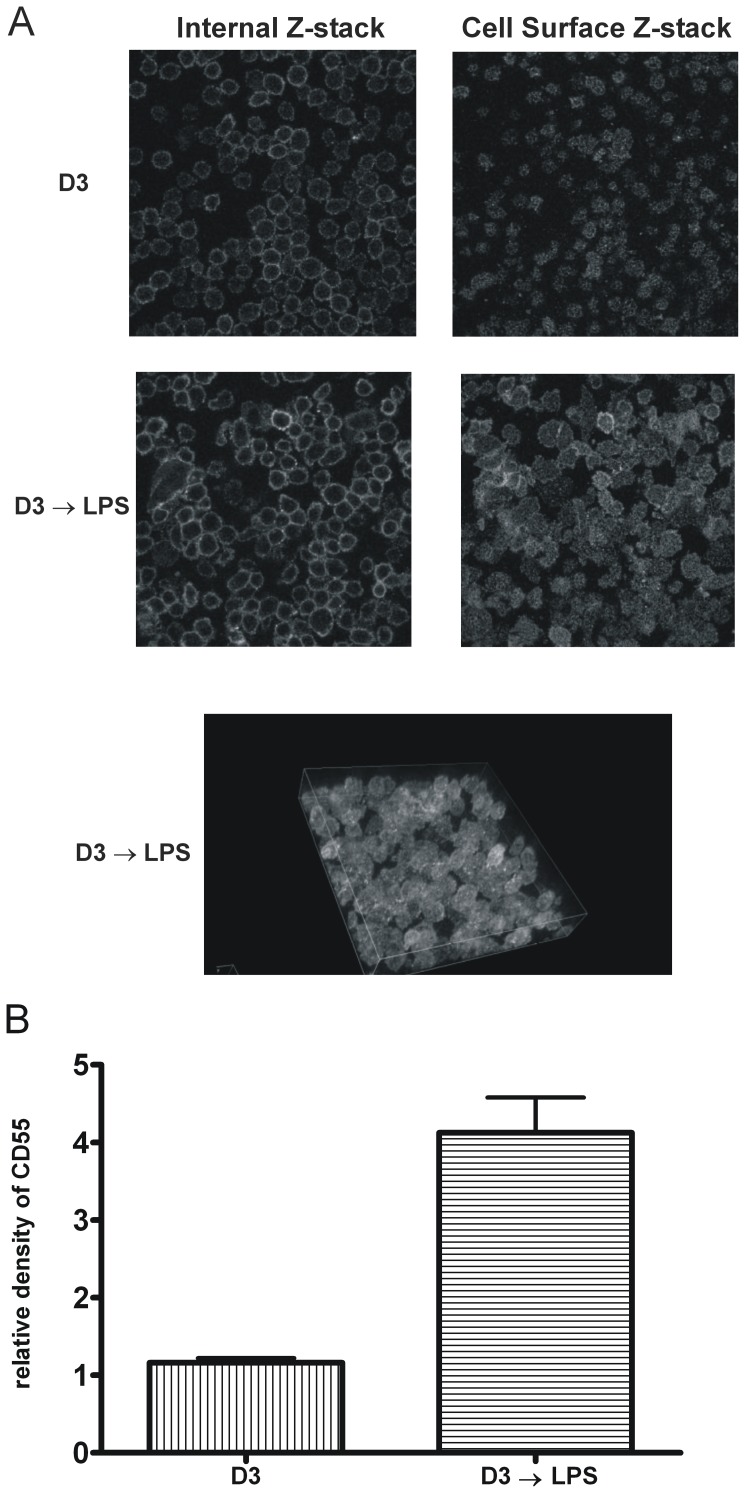
CD55 expression was enhanced on the surface of essentially all THP-1 cells at 24 h, following addition of LPS to 1,25-D3 pretreated cells. THP-1 cells were grown on coverslips, pretreated with 100 nM 1,25-D3 for 72 h and then stimulated with either vehicle or LPS for 24 h, as indicated. Serial 0.5 µm optical sections were obtained by confocal microscopy (40 × magnification). A, sections containing either a central (internal Z-stack) or the apical region of the cells (cell surface Z-stack) and the entire 3D image of LPS treated cells are shown. White represents CD55 staining; nucleic acid staining was omitted for clarity. B, shows a graphical representation of mean (±SD) CD55 expression levels (normalized to nucleic acid content) for three independent experiments with two fields quantified per experiment (n = 6).

A multiplexed fluorescence-based immunoblot (Fig. 3C) was used to determine LPS-induced protein expression of CD55, CD14 and CD11b in 1,25-D3-pretreated THP-1 cells. The results are graphically represented relative to β-actin in Fig. 3D. These data indicate that changes in CD55 protein expression directly correlate with the observed changes in mRNA pool size. Within 4 h, CD55 protein expression was elevated over 2-fold and the 4.9-fold increase observed after 24 h was essentially maintained throughout the 72 h time course. Since previous studies have reported that suboptimal signaling conditions can affect only a fraction of treated THP-1 cells [46], we used immunohistochemistry and confocal microscopy to establish the distribution profile of CD55 on intact non-permeabilized cells. Figure 4A shows two representative z-stacks (one mainly encompassing the cell surface, the other a mid-cell cross-sectional slice) of 1,25-D3-treated cells either before or after a 24 h exposure to LPS. The entire 3D image of the LPS-treated cells is shown in the lower panel of Fig. 4A (CD55 staining in white). These data demonstrate that essentially all cells uniformly increased cell surface CD55 expression in 1,25-D3 pretreated cells exposed to LPS for 24 h. A cell-permeant nucleic acid dye (omitted from the picture for clarity) was used to normalize expression, and the observed 4-fold increase in CD55 expression (Fig. 4B) was in agreement with the data from our immunoblot approach. Similar experiments were performed using controls only treated with LPS. Even though the 1,25-D3 untreated, non-adherent cells were only loosely fixed to the glass clover slip and the vast majority of them washed away during the procedure, we did not observe any difference in the level of CD55 expression between the subset of 1,25-D3 untreated cells that remained attached after staining and the subset of attached cells treated with LPS for 24 h (data not shown).

The protein expression profiles for CD14 and CD11b (Fig. 3C–D) did not correlate with the temporal changes in mRNA pool sizes. While the transient increase in CD14 mRNA pools size returned to (and was maintained at) control levels by the 24 h time point, CD14 protein expression continually increased by 6-, 12- and 21-fold at the 24, 48, and 72 h time points, respectively. The effect of LPS-induced expression of CD11b protein in 1,25-D3-primed cells was also at variance with changes in mRNA pool sizes. While the mRNA levels decreased more than 2-fold within 4 h of LPS challenge and were maintained at this lower level for 48 h, CD11b protein expression, which appeared to decrease in parallel with mRNA at 2 h, returned to preexposure levels by 4 h and was maintained at near control levels for the duration of the 72 h time course.

We propose that the disparity between mRNA pool size and the protein expression of 1,25-D3-pretreated THP-1 monocytic CD14 and CD11b in response to LPS insult, is likely due to additional post transcriptional regulatory mechanisms, such as microRNA and mTOR systems, both of which are important monocytic regulatory pathways that modulate mRNA stability and protein synthesis [47], [48]. Since our primary focus was on the regulated expression of CD55, we have not sought to investigate further the mechanism(s) by which CD14 and CD11b mRNA and protein pools are differentially regulated following LPS challenge in 1,25-D3-pretreated monocytes.

### LPS-stimulation of the Complement Cascade Regulatory Protein CD55 in 1,25-D3-pretreated THP-1 Cells is Directly Induced by NFκB Activation

**Figure 5:**
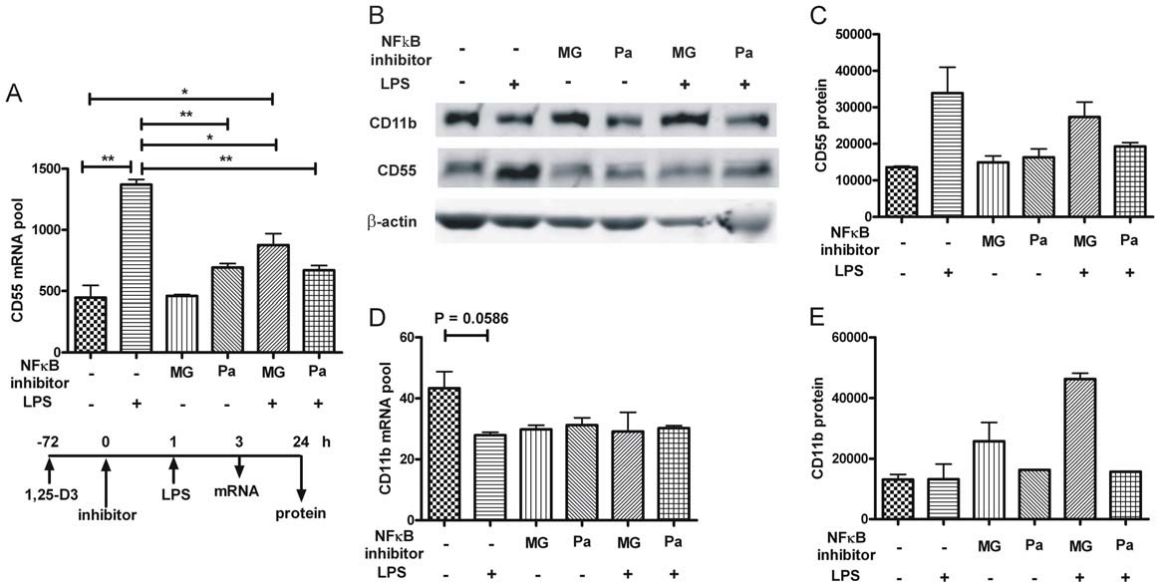
NF-κB activation was required for LPS-induced CD55 expression in 1,25-D3 pretreated THP-1 monocytes. As illustrated under panel A, at the end of the 72 h pretreatment phase with 100 nM 1,25-D3, either vehicle or one of two NF-kB inhibitors [50 µM MG132 (MG) or 30 µm parthenolide (Pa)] was added and the incubation continued for an additional 1 h. Then LPS (1 µg/ml) or vehicle was added. Cells were ultimately harvested at 2 h (mRNA) or 24 h (protein) post addition of LPS or vehicle. Quantitative PCR was used to determine CD55 (A) and CD11b (D) mRNA levels assessed at 2 h. Differences between mRNA pool sizes were determined using a two tailed student’s t test. Asterisks (Fig. 5A) indicate statistical differences between indicated treatments; *P<0.05, **P<0.01. CD55 and CD11b protein expression was assessed at 24 h following a vehicle or LPS challenge by immunoblotting (B) and quantification (C and E), respectively. Because of the semi-qualitative nature of the protein determinations, a statistical analysis was not performed on these data. Values represent means +/− SD (n = 3).

LPS-induced pro-inflammatory signaling activates the classic NF-κB pathway in THP-1 and primary monocytic cells [21]. Genomic wide studies in monocytic cell types recently identified the CD55 gene as a potential target for NF-κB regulation [22]. However, nothing is known about LPS regulation of CD55 in 1,25-D3-pretreated monocytes. Because available NF-κB inhibitors lack specificity, we used two different classes of inhibitors to test whether NF-κB activation was required for LPS-induced CD55 expression in THP-1 cells pretreated with 1,25-D3. With respect to NF-κB activation, MG132 is a 26S proteosome inhibitor that blocks NF-κB translocation into the nucleus by inhibiting the dissociation of NF-κB from the phosphorylated inhibitory IκB complex [49]. Parthenolide, a natural anti-inflammatory compound derived from the medicinal Feverfew herb, blocks NF-κB activation by directly binding to and inhibiting IκB kinase β [50]. For these studies, 1,25-D3-pretreated (72 h) THP-1 cells were exposed to vehicle, 50 µM MG132 (MG) or 30 µM parthenolide (Pa) for 1 h followed by the addition of LPS or vehicle for an additional 2 h (mRNA) or 24 h (protein). As shown in Figs. 5A–C, in 1,25-D3 treated THP-1 cells, MG132 partially inhibited subsequent LPS-induced CD55 expression relative to LPS-induced controls. MG132 alone had no affect on CD55 mRNA pool levels. Parthenolide completely blocked LPS-induced increases in CD55 mRNA pools (Fig. 5A) and had no appreciable affect on CD55 expression when administered alone (P = 0.085). Changes in CD55 protein expression (monitored 24 h after LPS challenge) were consistent with the observed effects of the inhibitors on mRNA pool sizes (Fig. 5B, quantified in Fig. 5C). These data strongly support the notion that NF-κB functionally regulates CD55 expression in 1,25-D3-pretreated cells.

We consistently observed an immediate decrease in CD11b mRNA pool size following an LPS challenge in 1,25-D3-treated THP-1 cells (see Fig. 2C). To determine if NF-κB played a direct role in the observed decrease in CD11b mRNA pools, inhibitor studies were performed using 1,25-D3 pretreated cells where the reduction in CD11b mRNA pool size was maintained for 48 h. The rapid decrease in mRNA pools following LPS challenge was not affected by MG132 or Pa pretreatment consistent with the notion that NF-κB does not directly influence the observed decrease in mRNA pool size (Fig. 5D). However, treatment with either MG132 or Pa alone resulted in a similar decrease in CD11b pool sizes. Only the P value for CD11b mRNA pools with or without LPS (panel D) is shown; a similar P value was determined with or without the inhibitors. Thus, the observed reduction in CD11b pool size does not appear to be a direct consequence of LPS-induced signaling. Moreover, MG132 either alone or in combination with subsequent LPS challenge, led to an increase in CD11b protein pool (Fig. 5B, quantified in Fig. 5E). We speculate that the apparent rapid protein turnover rate of CD11b reflects a mechanistic interrelatedness between phagocytosis of foreign particles and the involvement of the 26S proteosome in the processing and presentation of antigenic peptides [51].

In summary, the reduced levels of CD11b mRNA pools following LPS challenge in 1,25-D3 pretreated THP1 monocytes was not influenced by NF-κB activation. This is consistent with the fact that CD11b has not been identified as a NF-κB target gene in monocytes [22]. Our main conclusion from these studies was that 1,25-D3 pretreatment altered the responsiveness of the monocytic THP-1 cells to LPS insult and promoted the NF-κB-dependent enhanced expression of the cell surface regulator of complement activation, CD55.

## Discussion

The central observation of this study is that in human monocytic THP-1 cells, 1,25-D3 pretreatment altered the LPS-induced induction of the complement regulator CD55 from an immediate but transient effect to an immediate and sustained response pattern and that expression was dependent on NF-κB activation. Thus, the vitamin D3 system may play a role as an anti-inflammatory modifier of the host complement immune response [52]. Additionally, a coordinated control mechanism that enhanced CD55 expression and differentially regulated and modulated CD14 and the complement receptor 3 (CD11b/CD18) would contribute to an anti-inflammatory response by providing protection of monocytic-derived lineages actively engaged in pathogen recognition against complement-mediated cell lysis.

In its most basic form, the LPS signaling cascade in monocytic cell types principally involves the regulated activation of the bacterial molecule pattern receptor, the receptor proximal signal adaptor molecules and the inhibitor of κB (IκB) kinases. This signaling cascade leads to the modification and destruction of IκB proteins, thereby facilitating nuclear localization and recruitment of NF-κB to cognate target genes [53]. Attenuation of the NF-κB response occurs both through a negative feedback IκBα-dependent loop and by IκBα-independent mechanisms [19]. A recent global NF-κB target gene study meant to determine LPS-induced target genes in monocytic cells demonstrated that, prior to NF-κB activation, CD55 is populated with transcriptionally inactive p50 subunits, whereas LPS induces the rapid recruitment of the entire set (p65, c-Rel, RelB, p50 and p52) of NF-κB family members [22]. Our inhibitor studies strongly support the notion that LPS-induced NF-κB binding functionally modulates the transcriptional properties of the CD55 promoter. Moreover, to the best of our knowledge, our studies provide the first evidence that 1,25-D3 may modulate an anti-inflammatory response in human monocytic cell lineages by promoting a sustained NF-κB-dependent activation of an anti-inflammatory target gene. This contrasts with recent studies suggesting that the immunosuppressive action of 1,25-D3 in human monocytic lineages is directed, in part, by elevating the levels and enhancing the stability of IκBα [27] and/or suppressing RelB expression [28] and underscores the pleiotrophic immunosuppressive effects of 1,25-D3 [25].

Our data also suggest that 1,25-D3 indirectly modulates the transcriptional responsiveness of the CD55 promoter to LPS stimulation. We show that 1,25-D3 had no direct effect on the constitutive levels of CD55 expression within the first 24–48 h of treatment. However, during the later stages of exposure (72 h) mRNA and protein pools increased modestly by less than 2-fold. This increase in expression could be influenced by the recruitment of transcriptional regulators, such as C/EBPβ and MEF2D, whose expression are increased during the later stages of monocytic maturation [54] and/or by DNA and chromatin modifications within the CD55 promoter/enhancer regions [55]. These potential modifications to the transcriptional machinery of the CD55 gene could imprint NF-κB responsiveness. For example, in the context of 1,25-D3-induced changes in signaling networks and transcriptional regulators, this imprinting could block the attenuation of the transcriptional activation properties of NF-κB family members and/or enable NF-κB family members to initiate changes in the combination and/or modification state of transcriptional regulators, thereby increasing the production of CD55 mRNA’s. Additionally, enhanced expression of the LPS-binding proteins CD14 and complement receptor 3, both of which have been shown to influence the repertoire of LPS-inducible genes in mice macrophages [56], may modulate LPS-signaling pathways and contribute to the promotion of sustained NF-κB-dependent CD55 expression.

The observed partial inhibitory effect of MG132 (a proteosomal inhibitor that blocks NF-κB nuclear translocation by inhibiting IκB degradation) on LPS-induced CD55 expression suggests that additional non-NF-κB transcriptional regulators modulate CD55 expression. LPS-signaling in monocytic lineages is known to stimulate the MAPK pathway with subsequent activation of CREB [57], a known transcriptional activator of CD55 in epithelial cell lines [40]. Alternatively, activation of MAPK signaling initiated by other LPS sensing receptors, such as the G-protein coupled chemokine receptor 4 [58], could contribute to the modulation of CD55 expression. In contrast, parthenolide appeared to completely block the ability of LPS to influence CD55 mRNA pool size. Parthenolide was initially characterized as an anti-inflammatory agent that inhibited NF-κB activation by specifically binding to and inactivating IκB kinase β [50]; however, its specificity towards protein kinases has not been established. A more global inhibitory effect of parthenolide on cellular kinase activities, in particular those associated with the aforementioned alternative LPS-signaling pathways, would account for the more complete inhibition of LPS-induced CD55 expression. The direct correlation between mRNA pool size and CD55 protein expression levels suggest that the overall protein expression levels are not overtly influenced by post-transcriptional mechanisms.

The THP-1 cell line has been shown to be a reasonably accurate cell culture model system to explore monocytic-dependent immune responses [21] and appears to be well suited to study regulated expression of CD55. CD55 is expressed at potentially physiological relevant levels under normal growth conditions and the observed fold-induction is consistent with studies using endothelial and leukocytic primary cell cultures [23], [59]. We are currently investigating the interrelationship between 1,25-D3 and LPS-induced NF-κB signaling on sustained CD55 expression using normal human monocytes and on the identification of functional NF-κB recognition elements within the CD55 promoter region to test the effects of 1,25-D3 pretreatment on the dynamics of NF-κB family member binding.

During the course of these studies we also investigated the expression patterns of CD14 and CD11b, as they contribute to the sensitivity and coordinated inflammatory response of pathogen surveillance and in the phagocytosis of opsonized and non-opsonised particles during wound healing [11], [12], [14]. While the major thrust of our work was to investigate the regulated expression of CD55, we uncovered unanticipated features of CD14 and CD11b that are worth noting. First, as expected, both LPS and 1,25-D3 induced increases in CD14 and CD11b and the increase in mRNA pools correlated qualitatively with the protein expression shown herein and with previously reported flow cytometry analyses [39]. However, following 1,25-D3 pretreatment, LPS promoted a coordinated control mechanism that differentially regulated CD14 and CD11b mRNA and protein pools. In 1,25-D3 pretreated THP-1 cells, the mRNA pools for CD14 transiently increased less than 2-fold during the first 24 h of stimulation. However, protein levels continuously increased and were elevated over 21-fold after 72 h of LPS exposure. On the other hand, following LPS challenge, the mRNA pools of CD11b were reduced 2-fold, whereas protein pools transiently decreased but returned by 4 h to pre-treatment levels. While a mechanistic appreciation of these findings require further studies, post-transcriptional regulation is a key feature in monocytic responses, and systems such PI3K-AKT-mTOR [48] and/or microRNA/miRISC [60], [61] may play a role in the regulated expression of CD14 and CD11b. Additionally, the observed rapid fluctuation in CD11b protein pools following LPS challenge could reflect modulation of an intracellular protein pool and/or a rapid initialization and degradation of cell surface CR3. Additionally, we demonstrated that the 26S proteosomal inhibitor MG132 enhanced protein pools of CD11b, suggesting that the steady state levels of CD11b are modulated, in part, by the 26S proteosomal degradation pathway. Rapid targeting of phagocytosed particles to the 26S proteosomal system is consistent with its role in antigen presentation by monocytic cell lineages [49], [51]. The ability of the cell to rapidly modulate steady state CD11b levels may also have important roles in modulating leukocyte adhesion and trafficking during the early stages of an inflammatory insult [14] and in the modulation of innate immune pro-inflammatory response [12].

In summary, we demonstrated that 1,25-D3 influences monocytic responses to pro-inflammatory mediators, such as LPS, by an NF-κB-dependent activation of the complement regulatory protein CD55. Our studies provide the first evidence that 1,25-D3 may modulate an anti-inflammatory response in monocytic cell lineages by promoting a sustained NF-κB-dependent activation of anti-inflammatory target gene. Our data support the notion that the interrelationship between the vitamin D3 and complement systems should be explored in the pathophysiology of many human inflammatory and autoimmune diseases, such as rheumatoid arthritis, cardiovascular diseases [1], [62] and the pathophysiology of allograft rejection [2] and pregnancy [33]. The elevated and sustained expression profile mimics our recent findings on the expression profile of peripheral leukocyte CD55 in patients that exhibited PTL but did not progress to preterm delivery [18]. While the polarization and activation of monocyte cell lineages are complex processes influenced by the vitamin D3 hormonal system and by the spatial and temporal cascade of cytokine and chemokine induced signaling events, our study lends support to the notion that the vitamin D3 hormonal system may play a role in the pathophysiology of preterm labor. This may be particularly important when considering health disparity and the high rates of preterm births in African Americans, a population that exhibits reduced circulating vitamin D levels [63].
